# Medicinal Plants Used for the Traditional Management of Diabetes in the Eastern Cape, South Africa: Pharmacology and Toxicology

**DOI:** 10.3390/molecules23112759

**Published:** 2018-10-25

**Authors:** Samuel Odeyemi, Graeme Bradley

**Affiliations:** Department of Biochemistry and Microbiology, University of Fort Hare, Alice 5700, South Africa; sodeyemi@ufh.ac.za

**Keywords:** Ethnopharmacology, diabetes, medicinal plants, diabetes mellitus

## Abstract

The use of medicinal plants for the management of diabetes mellitus is on the rise in the developing countries, including South Africa. There is increasing scientific evidence that supports the claims by the traditional healers. In this review, we compare the families of previously reported anti-diabetic plants in the Eastern Cape by rating the anti-diabetic activity, mode of action and also highlight their therapeutic potentials based on the available evidence on their pharmacology and toxicity. Forty-five plants mentioned in ethnobotanical surveys were subjected to a comprehensive literature search in the available electronic databases such as PubMed, ScienceDirect, Google Scholar and Elsevier, by using “plant name” and “family” as the keywords for the primary searches to determine the plants that have been scientifically investigated for anti-diabetic activity. The search returned 25 families with Asteraceae highly reported, followed by Asphodelaceae and Alliaceae. Most of the plants have been studied for their anti-diabetic potentials in vivo and/or in vitro, with most of the plants having a higher percentage of insulin release and inhibition against carbohydrate digesting enzymes as compared with insulin mimetic and peripheral glucose uptake. Almost all the investigated plants also inhibit oxidative stress as part of their hypoglycemic activity with less toxicity. However, the isolation of their bioactive molecules is still lacking. This review provides a resource to enable thorough assessments of the therapeutic profiles of available medicinal plants used for the management of diabetes in the Eastern Cape, South Africa. Further studies such as the identification of the active ingredients of potent plants still need to be carried out; this may lead to new molecules in drug discovery and development.

## 1. Introduction

Diabetes mellitus is a disease associated with high blood sugar levels, a situation where the body is unable to effectively control the metabolism of glucose, the primary source of energy. It is described as a clinical syndrome characterised by inappropriate hyperglycemia caused by a relative or absolute deficiency of insulin or resistance to the action of the hormone at the cellular level [1]. This can be as a result of an auto-immune response where the immune system mistakenly attacks and kills the beta cells of the pancreas, thereby leading to the insufficient availability of insulin to regulate blood glucose levels (type I diabetes), or the cells become insensitive/resistant to the action of insulin (type II diabetes). The deficient or diminished effectiveness of endogenously synthesised insulin increases glucose concentration in the blood and urine.

Diabetes mellitus at the advanced stages affects other metabolic pathways of lipids, and manifests as hypercholesterolemia and hyperlipidemia, which are risk factors in atherosclerosis [2,3,4]. As the disease progresses, vital organs are affected such as the eyes, liver and kidneys, leading to retinopathy, increased gluconeogenesis, ketogenesis, diabetic ketoacidosis, non-ketotic syndrome, polyuria and nephropathy [5,6,7]. There is also an increase in the concentration of advanced glycation end products (AGEs), leading to the secondary complications of diabetes [7] such as impaired wound healing and foot ulcers, among others [8].

### 1.1. Aetiology of Diabetes Mellitus

To date, there is no apparent cure for diabetes mellitus type I, and therefore those with type I diabetes need to take insulin for life to control blood glucose levels. Diabetes mellitus type II is associated with the ingestion of high-calorie foods, family history of the disease, obesity, race, genetic disorders, smoking, inactivity, viral infections and drugs or chemicals, and can be managed with drugs and/or changed diet plans. Common symptoms of diabetes mellitus include frequent urination, excessive thirst, intense hunger and fatigue irritability, blurred vision, wounds that do not heal quickly or adequately, sexual dysfunction in men, and gum infections [9].

### 1.2. Prevalence

In recent times, there has been an increase in the prevalence of diabetes mellitus worldwide. A study in 2017 estimated that about 422–425 million adults were living with the disease and it was projected that this number would rise to 629 million adults (48% increase) affected with the disease by the year 2045 if necessary and adequate actions are not taken [10,11,12]. The African region has the highest proportion of undiagnosed diabetes, as investments, research, and health systems are slow to respond to this burden [13]. Available information suggests that diabetes is emerging as a significant health problem in Africa, including South Africa [13].

The Eastern Cape Province is estimated to have the highest percentage of poor residents (72.9%), in South Africa, with the majority of residents living in rural communities. The majority of these residents tend to rely on medicinal plants for the treatment of common diseases, including diabetes, because of their availability, affordability, effectiveness, and low side effects [14,15,16]. This review was therefore undertaken to identify which plants are used by traditional healers within the Eastern Cape province, South Africa, to treat diabetes.

It is imperative to continually reassess glycemic control in people with diabetes due to the progressive nature of the disease, which requires constant therapeutic regimen readjustment. Metformin (dimethylbiguanide), the preferred first-line oral blood-glucose-lowering agent to treat type II diabetes, comes from a derivate of French lilac *Galega officinalis* (also known as goat’s rue), suggesting that medicinal plants could be a rich source of anti-diabetic agents [17]. Several drugs such as biguanides and sulfonylurea which are presently employed in the management of diabetes have side effects such as worsening of heart disease, increased body weight and hypoglycaemia [18,19]. These side effects coupled with the high cost of anti-diabetic drugs has led to the search for plants with anti-diabetic properties and consequently their use in the management of diabetes [19,20].

### 1.3. Target Organs in Diabetes Treatment

**Figure 1:**
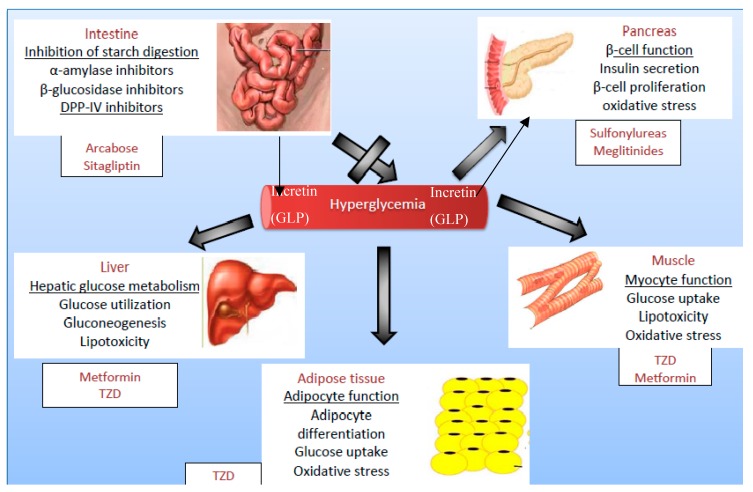
Summary of therapeutic targets for the management of diabetes mellitus. TZD = Thiazolidinedione. DPP-IV: Dipeptidyl peptidase IV; GLP: Glucagon-like peptide 1.

Most conventional and herbal treatments are targeted towards specific organs or metabolic pathways as shown in Figure 1. These treatments either activate chemicals that enhance insulin secretion or suppress hepatic glucose output. The potency of the documented medicinal plants used for the treatment of diabetes has been attributed to the presence of their phytochemicals. These phytochemicals are synthesised by plants to protect themselves from internal stresses such as free radicals, and external stresses from insects and pests; this property of plants explains their potential to cure diseases and their benefits in traditional medicine. The phytochemicals of these plants have been reported, and their mechanism of action has been suggested [21,22].

### 1.4. Ethnopharmacological Data

Forty-five plants identified from previous ethnobotanical surveys [15,23,24,25] for the management of diabetes in Eastern Cape Province, South Africa, were subjected to electronic searches in all University of Fort Hare subscribed databases.

## 2. Results and Discussion

Before selecting plants or families of plants to be included, priority was given to investigations carried out with samples collected from Eastern Cape, South Africa. Twenty-five families of the 45 plants were reported in ethnobotanical surveys (Table 1), although some of these plants have also been scientifically investigated elsewhere for their anti-diabetic properties to justify their traditional usage. Out of the 45 plants reviewed here, only three have been scientifically evaluated in vivo, 12 scientifically evaluated in vitro, and 12 have been evaluated both in vitro and in vivo, while 18 of them have not been scientifically evaluated for their hypoglycaemic effects, either in vivo or in vitro.

### 2.1. Ethno-Pharmacological Details of Plant Families with Documented Anti-Diabetic Activities

#### 2.1.1. Alliaceae (Three)

Three plants of the Alliaceae family have been reported for their antidiabetic properties in this family, namely *Allium sativum*, *Tulbaghia alliacea*, *Tulbaghia violacea*. *Harv.* [30]. *Allium sativum* is probably the most exploited in this family. Its hypoglycaemic, hypocholesterolaemic and hypotriglyceridaemic effects were studied in diabetic rats [31,32,33,34,35,36]. *Tulbaghia violacea*. *Harv.* was reported to improve glucose-stimulated insulin secretion (GSIS) in INS-1 pancreatic β-cells and glucose uptake in Chang liver cells [30]. There is very little scientific data on the anti-diabetic claim of *Tulbaghia alliacea*. The suggested mechanism of action of this family has been suggested to be pancreatic secretion of insulin, increasing the membrane potentials and *GLUT*-*2* expression in INS-1 cultured cells [30,37,38] while the allicin is reportedly mentioned as the leading bioactive molecule.

#### 2.1.2. Aloaceae (One)

*Aloe ferox Mill*, the only scientifically documented plant in this family for anti-diabetic properties, was investigated in streptozotocin (STZ)-induced type II diabetes rats and suggested that the potential for restoring hyperglycaemia could be through increased insulin secretion [39]. In a separate study, it was reported that the active molecules from *Aloe ferox Mill* are the phenolic acids/polyphenols, sterols, alkaloids, fatty acids, and indoles [40].

#### 2.1.3. Anacardiaceae (One)

*Sclerocarya birrea (A. Rich.) Hochst. subsp. caffra (Sond.) Kokwaro* is one of the frequently used medicinal plants in the Eastern Cape and has been extensively investigated for its anti-diabetic activities [24,41,42,43]. The α-amylase and α-glucosidase inhibitory activity have also been reported [36]. *Sclerocarya birrea* was reportedly associated with enhanced glucose metabolism by promoting the overall metabolic pathway of glucose metabolism that leads to ATP formation and enhanced glucose-stimulated insulin secretion in pancreatic β-cells [41,44]. The presence of medicinally-important molecules such as polyphenols, tannins, coumarins, flavonoids, triterpenoids and phytosterols were identified in the plant [42]. There is, however, a concern due to the in vitro toxicity results for *Sclerocarya birrea* [45].

#### 2.1.4. Apiaceae (One)

*Heteromorphica arborescens Hochst. Ex A. Rich.* was reported to be used in traditional medicine for the treatment of diabetes in the Eastern Cape, South Africa, but is yet to be scientifically investigated.

#### 2.1.5. Apocynaceae (Two)

*Catharanthus roseus* (L.) *G. Don.* and *Vinca major* L. were reported to possess anti-diabetic activity. The anti-diabetic property of *Catharanthus roseus* has been reported in both in vivo and in vitro studies [24], while only the in vitro study of *Vinca major* has been reported [26]. Vindolicine, an alkaloid, has been reported to be the most active anti-diabetic molecule in *Catharanthus roseus*, through it also has free radical scavenging capacity, enhanced glucose utilisation and PTP-1B inhibition [26,46]. While the mode of action of *Vinca major* has not been elucidated, in vitro toxicity results from the chronic use of *Catharanthus roseus* and *Vinca major* was reported, raising concern for its use in treating patients [26].

#### 2.1.6. Asphodelaceae (Five)

*Bulbine abyssinica*, *Bulbine natalensis (Syn. B. latifolia) MilL. Bulbine frutescens, Hypoxis hemerocallidea* and *Hypoxis colchicifolia Bak.* were reported in vitro, except for *Hypoxis colchicifolia* which has been studied both in vitro and in vivo [23,47,48,49,50,51]. It was suggested that the anti-diabetic molecules in *B. abyssinica* could be carvone, quercetin or psoralen [49]. The mode of hypoglycaemic action of *Bulbine abyssinica*, *Bulbine frutescens* and *Hypoxis colchicifolia* is yet unknown and still requires further studies, however, the hypoglycemic activities of *Hypoxis hemerocallidea* has been reported to be similar to that of metformin [50].

#### 2.1.7. Asteraceae (Thirteen)

Asteraceae is the most cited and documented family of medicinal plants in the traditional treatment of diabetes. However, not all have been scientifically investigated, and this includes *Artemisia afra Jacq.*, *Brachylaena discolour DC.*, *Brachylaena elliptica (Thunb.) DC*, *Conyza scabrida DC.*, *Helichrysum gymnocomum*, *Herichrysum nudifolium* L., *Herichrysum odoratissimum* L., *Herichrysum petiolare H* and *B.L.*, *Pteronia divaricata (P.J. Bergius)*, *Schkuhria pinnata (Lam.) Cabrera*, *Tarchonanthus camphoratus* L., *Vernonia amygdalina DeL.,* and *Vernonia oligocephala Sch. Bip*. The major mode of action of plants in this family has been reported to be through insulin release, repair of pancreatic β-cells, inhibition of carbohydrate digesting enzymes and oxidative stress [23,24,52,53,54,55,56,57,58,59,60,61,62,63]. Some of the anti-diabetic molecules isolated are saponins, flavanones, tannins and flavonoids (aglycones) [23,56,64,65,66].

#### 2.1.8. Buddlejaceae (One)

The only plant mentioned in this family is the *Chilianthus olearaceus Burch*. However, its anti-diabetic properties have not been investigated scientifically.

#### 2.1.9. Cannabaceae (One)

*Cannabis sativa* L. belongs to this family, and the in vivo anti-diabetic properties have been reported. Levendal and Frost [67,68] reported that the hypoglycemic effect could be through the increased energy utilisation or insulin release but in contrast, the in vitro study was not promising [38], therefore further studies are required to elucidate the mechanism of action of tetrahydrocannabinol and any other anti-diabetic molecules present in the plant.

#### 2.1.10. Caryophyllaceae (One)

*Dianthus thunbergii* is the only plant mentioned in this family [25]; however, there is a paucity of scientific data on its anti-diabetic properties.

#### 2.1.11. Celastraceae (Three)

Three plants were reported in this family; *Catha edulis (Vahl) Forrsk. ex EndL.*, *Elaeodendron transvaalense (Burtt Davy)* and *Lauridia tetragonia* [26]. Different parts of these plants have been reportedly used, including the leaf, stem, roots and bark. *Catha edulis* was effective in reducing blood glucose to normal in alloxan-induced diabetic rats, comparable to insulin, and was attributed to the presence of different chemical groups of molecules. This plant has insulinomimetic properties and inhibits carbohydrate digesting enzymes [26,69]. Dallak et al. and Saif-Ali [70,71], however, reported that the *Catha edulis* hypoglycemic activity was not significant in normal, glucose loaded and alloxan-induced diabetic rats. *Elaeodendron transvaalense* displayed hypoglycemic activities in three cell lines and also inhibits carbohydrate digesting enzymes [65]. It was therefore reported that phenolic molecules, elaeocyanidin, gallotannins and ouratea proanthocyanidin A isolated from *Elaeodendron transvaalense* are responsible for the anti-diabetic properties [29,72,73]. There is a paucity of scientific data on the anti-diabetic properties of *Lauridia tetragonia* in the literature.

#### 2.1.12. Cucurbitaceae (Two)

*Momordica balsamina L*. and *Momordica foetida Schumach.* were mentioned in this family for the management of diabetes in the family of Cucurbitaceae [26]. The extracts of both plants have been reported to be active in muscle cells, inhibiting carbohydrate digesting enzymes and prevention of oxidative stress [26,74,75]. Foetidin, isolated from *Momordica foetida*, has been reported to reduce blood glucose levels in normal but not in diabetic rats [70], however, these plants were toxic to cell lines [26].

#### 2.1.13. Ebenaceae (One)

The only plant reportedly used in this family is *Euclea undulata Thunb*. Molecules such as α-amyrin-3O-β-(5-hydroxy) ferulic acid, betulin, lupeol and epicatechin isolated from this plant have been reported for anti-diabetic activity in vitro [45,76]. The plant exhibits its anti-diabetic effects by insulin-dependent glucose uptake and inhibition of α-glucosidase [72].

#### 2.1.14. Fabaceae (One)

*Sutherlandia frutescens* L., the only plant reported in this family, has been investigated both in vivo and in vitro for its anti-diabetic activities [77,78,79]. *Sutherlandia frutescens* normalizes insulin levels and glucose uptake in peripheral tissues, suppresses intestinal glucose uptake, prevents insulin resistance and significantly reversed the effects of fructose and insulin on lipid accumulation in cell lines [80,81].

#### 2.1.15. Gentianaceae (One)

The anti-diabetic properties of *Chironia baccifera* L. was promising in vitro, reported by van de Venter et al. [26]. There are few scientific data of its anti-diabetic activity in animal models.

#### 2.1.16. Hyacinthaceae (Two)

*Albuca setosa (Jacq)* and *Albuca bracteata* (*Ornithogalum longibracteatum*) (*Jacq*) are reported in this family. The in vitro study showed glucose uptake in cell lines and inhibition of carbohydrate digesting enzymes. The mode of action is still not clear, but the insulinomimetic property has been suggested and prevention of oxidative stress. However, it has been suggested that the presence of saponins in *Albuca bracteata* could account for the anti-diabetic activity [23,82,83].

#### 2.1.17. Hypoxidaceae (Two)

*Hypoxis hemerocallidea Fisch.* and *C. A* and *Hypoxis argentae* of the family Hypoxidaceae have been reported to possess anti-diabetic properties. *Hypoxis hemerocallidea* caused significant reductions in the blood glucose concentration of the streptozotocin (STZ) induced diabetic rats by stimulating insulin release, promoting the cellular uptake and utilisation of glucose in the experimental animals [51,84]. Phytosterols and sterolin present have been implicated to be responsible for its anti-diabetic properties [85], while hypoxoside has been reported to be abundant in *Hypoxis* species. This will require further investigation to determine its anti-diabetic property [86].

#### 2.1.18. Lamiaceae (One)

The anti-diabetic properties of *Leonotis leonorus* have been reported to lower blood glucose in streptozotocin-induced diabetic rats and were compared to glibenclamide [87,88]. The mechanism of action of the plant has not been extensively investigated, but it was suggested to be its ability to potentiate insulin secretion from pancreatic beta cells or sensitizing insulin receptors. This plant was reportedly rich in phenolics and flavonoids [88].

#### 2.1.19. Loganiaceae (One)

*Strychnos henningsii* has been reported to induce hypoglycemic action in streptozotocin-induced diabetic rats and regularize complications in pathophysiological conditions associated with diabetes [89,90]. The in vitro anti-diabetic study also revealed glucose uptake in 3T3-L1 cells that was independent of Peroxisome Proliferator-activated Receptor γ (PPARγ) and inhibited the α-glucosidase enzyme [91]. Reports suggest that the plant is rich in phenols and alkaloids (*O*-acetylretuline) [92,93,94]. The mode of action was attributed to the ability to potentiate insulin secretion and protect pancreatic β-cells [90].

#### 2.1.20. Myrtaceae (One)

*Psidium guajava* L. was the only plant mentioned in this family used traditionally. The anti-diabetic properties have been reported in different animal models [95,96,97,98,99]. It was reported that the effective duration of *Psidium guajava* is less effective compared to metformin. However, the in vitro anti-diabetic properties were encouraging, as the aqueous root extract was active in fat and muscle cells and there was significant alpha-glucosidase inhibitory activity in the small intestine of diabetic mice [26,100,101,102,103]. It was suggested that the hypoglycaemic component might involve ursolic acid, oleanolic acid, arjunolic acid and glucuronic acid [103,104,105]. The antiglycation effect, an inhibitor of LDL glycation in both glucose and glyoxal induced models that were directly related to its polyphenolic content and free-radical scavenging ability, have also been speculated to be its mode of anti-diabetic action [103].

#### 2.1.21. Menispermaceae (One)

*Cissampelos capensis* L.f. was the only plant mentioned in this family and has been reported to be rich with alkaloids and flavonoids [25,94]. There are few reports of the hypoglycemic activity in animal models in the literature. However, the reports of van de Venter et al. [26] suggested that the glucose uptake in Chang cells is encouraging.

#### 2.1.22. Portulaceae (One)

*Anacampseros ustulata* was the only plant mentioned in this family [25]. There is limited information on the scientific study of its anti-diabetic properties.

#### 2.1.23. Rutaceae (One)

*Ruta graveolens* L. was the only plant mentioned in this family [28]. This plant has been investigated both in vitro and in vivo for its anti-diabetic properties [23,24,106]. The mechanism of action of this plant has been reported to be through their insulinogenic effects by improving peripheral insulin action, enhancing peripheral glucose uptake, inhibition of intestinal glucose and cholesterol absorption, affecting mediators of insulin resistance, decreasing hepatic glucose output and ameliorating oxidative stress [106].

#### 2.1.24. Solanaceae (One)

*Solanum aculeastrum* was the only plant mentioned in this family by the traditional healers in the Eastern Cape [25]. There is limited information on the scientific study of its anti-diabetic properties. 

#### 2.1.25. Xanthorrhoeaceae (One)

*Bulbine frutescens* L. (*Willd*) was the only plant mentioned in this family [15]. *Bulbine frutescens* was reported to increase glucose utilization in Chang cells similar to the response observed for *Ornithogalum longibracteatum* and knipholone [23]. However, there is no scientific information on the in vivo studies of its anti-diabetic properties

### 2.2. Pharmacological Evidence

#### 2.2.1. Bioactive Molecules

The potency of the documented medicinal plants used for the treatment of diabetes has been attributed to the presence of their phytochemicals. This property of plants explains their potential to cure diseases and their benefits in traditional medicine [107]. In this review, the different groups of molecules identified have either been fractionated through assay-guided isolation or based on the abundant presence in these plants as summarised in Table 2. However, few data are available on the specific bioactive molecules responsible for the anti-diabetic activities of the investigated plants. The following are the phytochemicals reported in the families of plants investigated for their anti-diabetic activity in the Eastern Cape.

##### Phenolic Molecules

Phenolic molecules are a large group of molecules reported to possess anti-diabetic activities. Seven families were mentioned to contain phenolic molecules as their active principle and responsible for the anti-diabetic activity. This may be in part due to their established antioxidant activity and prevention of advanced glycated end product (AGE) formation. Therefore, phenolic molecules can ameliorate complications associated with diabetes involving high oxidative stress conditions such as retinopathy, atherosclerosis, neuropathy and nephropathy [125,126]. Phenolics have also been reported to protect the pancreatic cells from oxidative stress, hence regenerating the β-cells and improving insulin sensitivity [107]. Therefore, it can be suggested that a plant rich in phenolics will be a good candidate for the management of diabetes or amelioration of its complications. 

##### Terpenes

This group of molecules were mentioned in five families of plants (Table 2). Several molecules in this group have been identified or isolated. Examples of these are triterpenes such as α-amyrin-3O-β-(5-hydroxy) ferulic acid, terpenoids, sesquiterpenoids and sesquiterpene. These molecules have been suggested to potentiate their anti-diabetic activity through the stimulation of insulin secretion [45,127]. Hence, isolation of terpenes for anti-diabetic activities could be directed toward the potentiation of insulin secretion of the pancreas.

##### Saponins

Saponins were mentioned in five families of plants and have been characterised by their bitter taste. It is not known how saponins elicit their anti-diabetic properties, but evidence from the family of plants reported to have saponins suggests that they may be involved in insulin secretion [34,88,103].

##### Alkaloids

Alkaloids are the most mentioned group of molecules, found in nine families of plants used for the management of diabetes in the Eastern Cape. This can be attributed to their vast medicinal properties such as antioxidant, inhibition of carbohydrate digesting enzymes, enhancement of glucose uptake in cells and enhancement of insulin release. Alkaloids such as hypoglycin, mahanimbine, vindoline I, vindolidine II, vindolicine III and vindolinine IV have been reported for their anti-diabetic activities [34,46,88,112].

##### Hydroxylated Molecules Including Sugars

This group of molecules are the least mentioned and encompass other types of molecules other than the groups above. This group includes cardiac glycosides, sterols, fatty acids, ursolic acid, oleanolic acid, arjunolic acid, phytosterols, sterolin, glucuronic acid, betulin, lupeol, epicatechin and indoles [85,104,121].

#### 2.2.2. In Vitro Investigation of Hypoglycaemic Activity

It is essential to investigate the toxicity and anti-diabetic activities of these plants in vitro to ascertain the mechanism before in vivo studies because some hypoglycemic activities observed in plants are a side effect of their toxicity [125]. More than 50% of the plants reported here have been investigated for their anti-diabetic activities in vitro, such as the inhibition of carbohydrate digesting enzymes and glucose uptake in mammalian cell lines.

#### 2.2.3. In Vivo Investigation of Hypoglycaemic Activity

The aetiology of diabetes is characterized by high blood glucose, hence, in vivo studies monitor the lowering effect of blood glucose. It has been reported that even though most plants were investigated in the type I diabetic model in experimental animals, some are also effective as hypoglycemic agents for type II diabetes [125]. In this review, only 15 plants have been evaluated in vivo. These plants have been shown to increase insulin secretion, interact with insulin receptors, activate the PPARγ receptor or ameliorate complications of diabetes as their mechanism of action [34,36,46,110].

#### 2.2.4. Dosages

One of the long-time challenges of medicinal plant usage is the dosage. Therefore, most of the doses of the plants used in vitro and in vivo were based on the cytotoxicity or acute and sub-acute experiments. Most of the in vitro investigations were carried out at doses between 10–50 µg/mL. For instance, the in vitro investigation of *Tulbaghia violacea* was carried out at a maximum dose of 50 µg/mL for INS cells and 10 µg/mL for C2C12, Chang liver cells and 3T3-L1 cells [30]. *Catharanthus roseus* was investigated at a maximum dose of 12.5 µg/mL in Chang liver cells. However, reports suggest that *Bulbine frutescens* at 50 µg/mL increases glucose utilization more than insulin in C2C12 cells, and promotes glucose uptake at a dose of 12.5 µg/mL in Chang liver cells [23]. Furthermore, in vivo investigations were carried out at dose ranges between 300–800 mg/kg body weight. *Allium sativum* given to human subjects at 300 mg thrice daily and metformin at 500 mg twice daily showed a similar reduction in blood glucose [34].

### 2.3. Toxicological Evidence

The evaluation of the safety and toxicity of the reported medicinal plants is highly imperative due to the availability, affordability and widespread belief of the acceptability. In fact, one of the main reasons for hesitance against the use of medicinal plants and herbal products into the health care system by healthcare practitioners is the toxicity concern. There is limited information available in the literature on the potential toxicity or mutagenicity resulting from the long-term use of these families of plants. It is therefore necessary to screen these plants for toxicity or mutagenicity to differentiate toxic effects from pharmacological efficacy [125]. The toxicity of some species of certain families has been reported to cause certain discomforts such as abdominal pain, gastroenteritis, cessation of gastrointestinal peristalsis, contraction of the pupils and sloughing of the intestinal mucosa [103,128]. Members of the families of Alliaceae, Anacardiaceae, Apocynaceae, Asteraceae, Buddlejaceae, Cucurbitaceae, Hyacinthaceae, and Xanthorrhoeaceae have been reported to be cytotoxic in vitro [15,23,82,124]. The traditional knowledge of the use of a plant is necessary where there is no scientific evidence to ascertain its safety. Asteraceae has been reported to synthesise pyrrolizidine alkaloids that serve as a defence against insects; these alkaloids are known to be hepatotoxic, although these alkaloids have not been mentioned in any of the plants under investigation. However, they must be used with caution. In some cases, the hypoglycemic agent identified in the plant could also be toxic, such as tetrahydrocannabinol from *Cannabis sativa* and to some extent vindolicine from *Catharanthus roseus* [46,129].

Another aspect of safety concern is the potential herbal–drug interaction. The knowledge of the synergistic effect between the herb and drug can be harnessed towards dose adjustments that otherwise could be detrimental if not appropriately monitored and evaluated. The interaction could be pharmacodynamic if the phytochemicals present in the plant modify the pharmacological effect of a drug as a result of its biochemical or physiological effect on the body, or pharmacokinetic if the herb interacts with the administration, distribution, metabolism or excretion (ADME) of a drug, which invariably could affect the fate/bioavailability of the co-administered drug. The herb–drug interaction could modulate the activity of xenobiotic metabolizing enzymes and/or various drug transporters [125,130]. An example of a herb–drug interaction is *Allium sativum*, which enhances the pharmacological effect of anticoagulants such as warfarin or fluindione and reduces the efficacy of anti-retroviral drugs such as saquinavir [131]. On the other hand, co-administration of *Allium sativum* with metformin improved glycemic control considerably [32].

## 3. Conclusions

Almost all the families of plants that have been scientifically investigated in the Eastern Cape of South Africa for the management of diabetes mellitus have more than one mechanism of action. The mechanisms of action of *Tulbaghia violacea*, *Catharanthus roseus* and *Bulbine frutescens* were investigated at doses between 10–50 µg/mL using several cell lines, including INS, C2C12, Chang liver and 3T3-L1 cells, while in vivo investigations were carried out in human subjects at dose ranges between 300–800 mg/kg body weight, using *Allium sativum* with metformin as control. Nine of the families were reported to potentiate insulin release, seven inhibit carbohydrate digesting enzymes, four are insulinmimetic in action, three inhibit oxidative stress or scavenge free radicals, one increases the expression of GLUT-2, while only one was reported to be similar to metformin.

From this review, five families of plants have not been scientifically investigated, or the mechanism of action is not yet known. It is also worthy to note that many of the bioactive molecules of these plants are yet to be isolated and clinically investigated.

Therefore, considering the simultaneous increase in the prevalence of diabetes and the traditional management, great effort needs to be invested in the isolation and purification of these bioactive molecules, determination of the mechanism of action and comparison of their activity against the existing conventional drugs.

## Figures and Tables

**Table 1:** Ethnobotanical information of plants used by traditional healers in Eastern Cape, South Africa. S/N: Serial number.

S/N	Family	Plants	References
1	Alliaceae	*Allium sativum*	[25]
		*Tulbaghia alliacea*	[25]
		*Tulbaghia violacea. Harv.*	[23]
2	Aloaceae	*Aloe ferox Mill*	[25]
3	Anacardiaceae	*Sclerocarya birrea (A. Rich.) Hochst. subsp. caffra (Sond.) Kokwaro*	[26]
4	Apiaceae	*Heteromorphica arborescens. Hochst. Ex A. Rich.*	[15,24]
5	Apocynaceae	*Catharanthus roseus* (L.) *G. Don.*	[15,26]
		*Vinca major* L.	[26]
6	Asphodelacea	*Bulbine abyssinica*	[25]
		*Bulbine natalensis*	[25]
		*Hypoxis colchicifolia Bak.*	[15]
7	Asteraceae	*Artemisia afra Jacq.*	[15,27]
		*Brachylaena discolor DC.*	[15,24]
		*Brachylaena elliptica (Thunb.) DC*	[28]
		*Brachylaena ilicifolia*	[15]
		*Conyza scabrida DC.*	[24,27]
		*Helichrysum gymnocomum*	[25]
		*Herichrysum nudifolium* L.	[15]
		*Herichrysum odoratissimum* L.	[15]
		*Herichrysum petiolare H & B.L.*	[15,24]
		*Tarchonanthus camphoratus* L.	[23]
		*Vernonia amygdalina DeL.*	[15]
		*Vernonia oligocephala Sch. Bip.*	[15,27]
8	Buddlejaceae	*Chilianthus olearaceus Burch.*	[15]
9	Cannabaceae	*Cannabis sativa* L.	[26]
10	Caryophyllaceae	*Dianthus thunbergii*	[25]
11	Celastraceae	*Catha edulis (Vahl) Forrsk. ex EndL.*	[26]
		*Lauridia tetragonia*	[25]
12	Cucurbitaceae	*Momordica balsamina* L.	[26]
		*Momordica foetida Schumach.*	[26]
13	Ebenaceae	*Euclea undulata Thunb.*	[29]
14	Fabaceae	*Sutherlandia frutescens* L.	[24,28]
15	Gentianaceae	*Chironia baccifera* L.	[26]
16	Hyacinthaceae	*Albuca setosa*	[25]
		*Ornithogalum longibracteatum (Jacq)*	[23]
17	Hypoxidaceae	*Hypoxis argentae*	[24,25]
		*Hypoxis hemerocallidea Fisch.* and *C. A*	[15]
18	Lamiaceae	*Leonotis leonorus*	[24,25,27]
19	Loganiaceae	*Strychnos henningsii*	[25]
20	Menispermaceae	*Cissampelos capensis L.f.*	[25,26]
21	Myrtaceae	*Psidium guajava* L.	[26]
22	Portulaceae	*Anacampseros ustulata*	[25]
23	Rutaceae	*Ruta graveolens* L.	[23,27,28]
24	Solanaceae	*Solanum aculeastrum*	[25]
25	Xanthorrhoeaceae	*Bulbine frutescens* L. *(Willd)*	[23]
		*Bulbine natalensis (Syn. B. latifolia) MilL.*	[15]

**Table 2:** Ethno-pharmacological details of plant families used by traditional healers in Eastern Cape, South Africa. PI3K: Phosphatidylinositol-3 kinase; MAPK: mitogen-activated protein kinase PPARγ: Peroxisome Proliferator-activated Receptor γ, PPARα: Peroxisome Proliferator-activated Receptor α and PPARδ: Peroxisome Proliferator-activated Receptor δ.

	Family	Bioactive Molecules	Toxicity	Mechanism of Action	References
1	*Alliaceae*	Allicin, tannins, cardiac glycosides, saponins, alkaloids	Some fatalities including abdominal pain, gastroenteritis, cessation of gastrointestinal peristalsis, contraction of the pupils and sloughing of the intestinal mucosa have been implicated in some members	Pancreatic secretion of insulin	[34,36,37,108]
2	*Aloaceae*	Phenolic acids/polyphenols, sterols, alkaloids, fatty acids, and indoles	Not known	Antioxidant	[39,40,57,109]
3	*Anacardiaceae*	Polyphenols, flavonoids, saponins /saponides, triterpenes, tannins, alkaloids, steroids and cardiac glycosides.	Mixed results for toxicity, not cytotoxic to the C2C12, 3T3-L1 and HepG2 cells and in rat models. Serious concern from the in vitro toxicity results for *Sclerocarya birrea.*	Increase glucose absorption, possesses insulin-mimetic properties, inhibition of α-amylase and α -glucosidase and interactions with the insulin receptor that lead to the activation of biochemical cascades (PI3K and MAPK)	[110,111,112]
4	*Apiaceae*	Not known	Not known	Not known	
5	*Apocynaceae*	Alkaloids	*Catharanthus roseus* and *Vinca major* are cytotoxic in vitro	Enhance glucose utilization and PTP-1B inhibition, activation of PPARγ, PPARα and PPARδ. Good antioxidants	[26,46,113]
6	*Asphodelacea*	Phenolics and aloe emodin	Not known	Decrease hepatic glucose production similar to metformin	[50]
7	*Asteraceae*	Saponins, flavanones, tannins, flavonoids (aglycones), aesquiterpenoids, sesquiterpene lactones, alkaloids and polysaccharide, bisabolene	Cytotoxicities at higher concentrations have been reported	Insulin release, repair of pancreatic β-cells, inhibition of carbohydrate digesting enzymes and oxidative stress	[23,56,64,65,66,114,115]
8	*Buddlejaceae*	Not known	Toxic molecules have been isolated from plants in this family	No scientific information about the anti-diabetic properties	[24,116]
9	*Cannabaceae*	Not known	Not known	Insulin release	[26,67,68,117]
10	*Caryophyllaceae*	Not known	Not known	Not known	
11	*Celastraceae*	Phenolic molecules, elaeocyanidin, allotannins, ouratea proanthocyanidin A and triterpenes	Not known	Insulinomimetic properties and inhibits carbohydrate digesting enzymes	[26,29,69,72,73,118,119]
12	*Cucurbitaceae*	Glycosides, globulins, alkaloids, triterpenoids and phenolic molecules	Cytotoxic to cell lines	Insulinomimetic properties; inhibit carbohydrate digesting enzymes and prevention of oxidative stress	[26,120,121,122]
13	*Ebenaceae*	α-amyrin-3O-β-(5-hydroxy) ferulic acid, betulin, lupeol and epicatechin	Not known	Insulin dependent glucose uptake and inhibition of α-glucosidase	[45,76]
14	*Fabaceae*	Phenolic, flavonoids	Not known	Normalizes insulin levels, glucose uptake in peripheral tissues suppresses intestinal glucose uptake, prevents insulin resistance and significantly reversed the effects of fructose and insulin on lipid accumulation	[51,78,123]
15	*Gentianaceae*	Not known	Not known	Not known	[26]
16	*Hyacinthaceae*	Alkaloids, saponins, polyhydroxylated pyrrolidines, piperidines, (2R,5R)-bis(dihydroxymethyl)-(3R,4R)-dihydroxypyrrolidine (DMDP) and 1,4-dideoxy-1,4-imino-d-arabinitol (d-AB1)	Some members are highly toxic	Glucose uptake in cell lines and inhibition of carbohydrate digesting enzymes	[23,82,107,124]
17	*Hypoxidaceae*	Phytosterols and sterolin	Reported to be toxic only at high doses (≥1800 mg/kg)	Stimulating insulin release	[85]
18	*Lamiaceae*	Tetracyclic triterpenoid, carbohydrates, alkaloids, flavonoids,	*L. leonurus* has been reported to be toxic in rats	Insulin secretion	[92]
		tannins, steroids, terpenes/triterpenes and saponins			
19	*Loganiaceae*	Phenols and alkaloid (*O*-acetylretuline)	Some of the genus in this family e.g., *Strychnos* are extremely toxic, producing the poisin strychnine	Potentiate insulin secretion	[25,26,90,91,94]
20	*Menispermaceae*	Alkaloids and flavonoids	Not cytotoxic	Glucose uptake in adipocytes	[25,26,94]
21	*Myrtaceae*	Polyphenolics, ursolic acid, oleanolic acid, arjunolic acid and glucuronic acid	Not known	Free radical scavenging, alpha-glucosidase inhibitory activity	[103,104,105]
22	*Portulaceae*	Not known	Not known	Not known	
23	*Rutaceae*	Not known	Not known	Insulin action, inhibition of intestinal glucose uptake	[106]
24	*Solanaceae*	Not known	Not known	Not known	
25	*Xanthorrhoeaceae*	Not known	Cytotoxicity reported	Increase glucose utilization in Chang cells	[15,23]
